# Functional and morphological changes of the retinal vessels in Alzheimer’s disease and mild cognitive impairment

**DOI:** 10.1038/s41598-018-37271-6

**Published:** 2019-01-11

**Authors:** Giuseppe Querques, Enrico Borrelli, Riccardo Sacconi, Luigi De Vitis, Letizia Leocani, Roberto Santangelo, Giuseppe Magnani, Giancarlo Comi, Francesco Bandello

**Affiliations:** 1 Ophthalmology Department, San Raffaele University Hospital, Milan, Italy; 2 Ophthalmology Clinic, Department of Medicine and Science of Ageing, University G. D’Annunzio Chieti-Pescara, Chieti, Italy; 3 Department of Neurology and Institute of Experimental Neurology-INSPE, San Raffaele University Hospital, Milan, Italy

## Abstract

Imaging and histopathological studies have demonstrated that structural changes of the retina affect subjects with Alzheimer’s disease (AD) or mild cognitive impairment (MCI). The aim of this study was to quantitatively investigate the retinal vessels in these disorders, using dynamic vessel analyzer (DVA) and optical coherence tomography angiography (OCTA) analysis. Twelve subjects with AD, 12 subjects with MCI, and 32 gender- and age-matched controls were prospectively enrolled. Mean ± SD age was 72.9 ± 7.2 years in the AD group, 76.3 ± 6.9 years in the MCI group, and 71.6 ± 5.9 years in the control group (p = 0.104). In the DVA dynamic analysis, the arterial dilation was decreased in the AD group (0.77 ± 2.06%), in the comparison with the control group (3.53 ± 1.25%, p = 0.002). The reaction amplitude was decreased both in AD (0.21 ± 1.80%, <0.0001) and MCI (2.29 ± 1.81%, p = 0.048) subjects, compared with controls (3.86 ± 1.94%). OCTA variables did not differ among groups. In the Pearson correlation analysis, amyloid β level in the cerebrospinal fluid was directly correlated with the arterial dilation (R = 0.441, p = 0.040) and reaction amplitude (R = 0.580, p = 0.005). This study demonstrate that Alzheimer’s and MCI subjects are characterized by a significant impairment of the retinal neurovascular coupling. This impairment is inversely correlated with the level of amyloid β in the cerebrospinal fluid.

## Introduction

Alzheimer’s disease (AD) is the most common form of neurodegeneration among older individuals in developed countries, with an estimated prevalence of 36.5 million^1^. This form of dementia is clinically characterized by severe cognitive decline and socio-behavioral manifestations^1^. Furthermore, subjects with AD may experience visual impairment, including narrowed visual field, dyschromatopsia, deficits in contrast sensitivity, abnormal eye movements, and alteration in visual-evoked potentials^2^.

Alzheimer’s disease is preceded by a variable transitional phase, which affects older subjects with heterogeneous cognitive and functional impairment, the latter not crossing the threshold for dementia. This condition has been termed “mild cognitive impairment” (MCI) and the affected patients have an increased risk of developing dementia, especially AD^3,4^.

AD is a complex disease with multifactorial etiologies. Many factors have been implicated in the pathogenesis and progression of this disorder, the earliest and most important is known to be a continuum of detrimental processes, most likely beginning with the accumulation of misfolded amyloid β-protein (Aβ) and hyperphosphorylation of tau proteins, which shape neurofibrillary tangles^5^. This accumulation of debris is thought to initiate a cascade of secondary processes, which involve inflammation, oxidative stress, vascular abnormalities, and neuronal loss^6,7^. Of note, small vessel disease of the brain has been demonstrated to be implicated in AD and MCI pathogenesis^8^. Furthermore, these neurodegenerative disorders are associated with early neurovascular dysfunction, which leads to an altered blood flow regulation and contributes to disease pathogenesis^9,10^.

Although cerebral small vessel disease has thus been implicated in the development of AD and MCI, the cerebral microcirculation remains difficult to investigate *in vivo*. Since the cerebral and retinal vasculature have similarities regarding embryological origin, anatomical features and physiological properties^11^, investigating the retinal vessels may thus be valuable in providing new insights into the pathogenesis of Alzheimer’s disease and mild cognitive impairment. Furthermore, the morphological and functional characteristics of the retinal vessels might be useful as indicators of disease progression.

Of note, some reports have already shown that retinal vascular alterations may be present in eyes of subjects with AD or MCI^12^. Nevertheless, all these descriptions have been made using fundus photographs. The introduction of new imaging modalities has however revolutionized retinal imaging. These techniques allow topographic qualitative investigation and quantitative measurements of vessels at various depth-resolved levels of the retina.

Recent studies have investigated the retinal microvascular networks in normal and pathologic states using dynamic vessel analyzer (DVA) and optical coherence tomography angiography (OCTA) devices in adults and children^13–17^.

However, to the best of our knowledge, detailed description of the functional and morphological characteristics of the macular microvasculature in AD and MCI subjects has not been performed. The aim of this study was thus to explore retinal vessels in subjects affected by AD or MCI, using DVA and OCTA analysis.

## Methods

### Study participants

This study is a prospective, observational, case-control study. Subjects with a clinical confirmed diagnosis of Alzheimer’s disease or mild cognitive impairment were enrolled from the ophthalmology and neurology departments at the San Raffaele Hospital in Milan, Italy. The study was approved by the San Raffaele Institutional Review Board and adhered to the tenets of the Declaration of Helsinki and Health Insurance Portability and Accountability Act. Written informed consent was obtained from all subjects prior to enrollment in the study.

Before the enrollment, all affected subjects underwent a standardized dementia assessment, which included: (i) Mini-Mental State Examination (MMSE), (ii) medical history collection, (iii) physical and neurological examination, (iv) neuropsychological testing, (v) electrocardiogram, (vi) electroencephalography, (vii) blood tests (VDRL serologic screening tests for syphilis, thyroid function, vitamin B12, folate and homocysteine), and (viii) either standard (computed tomography and/or magnetic resonance imaging) or functional neuroimaging with positron emission tomography with 2-deoxy-2-[fluorine-18]fluoro- D-glucose (^18^F-FDG PET), in order to rule out other potential causes accounting for cognitive decline. Moreover, quantification of cerebrospinal fluid (CSF) biomarkers (Aβ, tau protein, and phosphorylated tau protein) was performed in all subjects. Of note, the CSF biomarkers were considered positive in agreement to the international normative criteria: Aβ42 was higher than 500 ng/L^18^, t-tau protein was lower than 350 ng/L^19^, and phosphorylated tau protein was lower than 61 ng/L^20^. Finally, the diagnosis of AD and MCI was made following the NIA-AA criteria^21,22^. Exclusion criteria were the presence of any other neurologic or psychiatric disorders, including suspicion of other types of dementia.

Assuming age may affect retinal and vascular parameters^23^, a control group matched for age was also included in the current analysis. Control subjects were volunteers with no evidence of cognitive impairment (based on the MMSE assessment) or neurological/psychiatric disorder.

Exclusion criteria for either patient and control group were: (i) history of diabetes mellitus, uncontrolled hypertension, heart disease, or other serious chronic medical conditions; (ii) significant media opacity; (iii) evidence of optic nerve or retinal disease, as evaluated by dilated fundus examination and OCT/OCTA; (iv) refractive error greater than 6 diopters (D); and (v) pharmacologic pupillary dilation lower than 6 mm.

All AD/MCI subjects and controls were enrolled between May 2016 and May 2017 and received a complete ophthalmologic examination, which included the measurement of best-corrected visual acuity (BCVA), intraocular pressure, and dilated ophthalmoscopy. BCVA measurements were made using a Snellen chart and were converted to the logarithm of the minimum angle of resolution (LogMAR). Finally, either pathological and healthy control group underwent DVA, OCTA, and OCT imaging.

### Dynamic vessel analyzer

The DVA (DVA; Imedos Systems UG, Jena, Germany) is a commercially available system which is composed of a fundus camera, video camera, real-time monitor, and a personal computer with analysis software.

The DVA examination has been described in depth in previous reports^13,14,24,25^. In brief, this device tests the retinal vessel changes in response to diffuse luminance flicker (dynamic analysis). During the examination, the subject focuses into the retinal camera in order to allow the examiner to visualize the subject’s fundus with a red-free light. An arteriolar and venular segments are thus selected and their changes in diameter are recorded and measured at baseline and during/after a flickering-light stimulation. Of note, this stimulation lasts 20 seconds and then the previously selected vessels are measured for 80 seconds. This cycle of stimulation and measurements was repeated twice in each eye, with a total duration of the examination of 350 seconds per eye. Importantly, the system works by automatically suspending the test when subjects blink or move their eyes.

**Figure 1:**
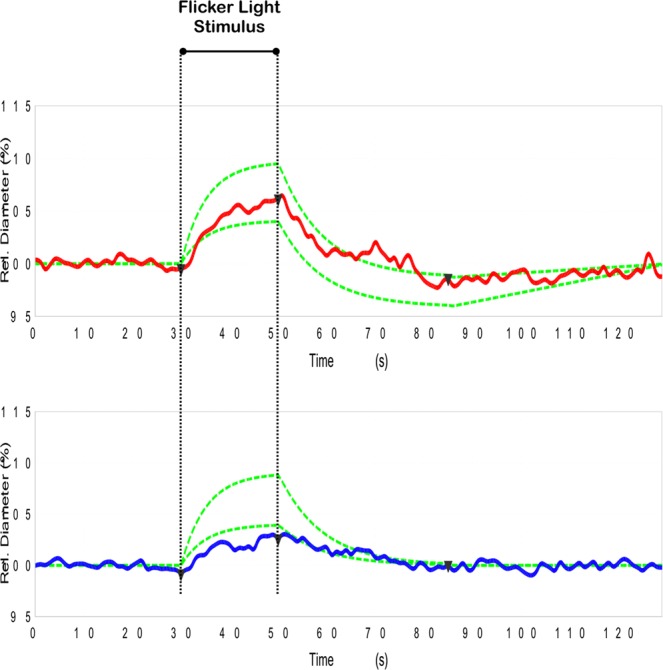
Response curves to flicker light stimulus from a healthy subject. The example of a healthy control subject shows a characteristic response curve to flicker light stimulus (red for arterial and blue for vein) after baseline diameter acquisition. With intact autoregulation, a characteristic primary vasodilation and secondary vasoconstriction is observed. The green lines define the area in which a normal curve is expected to fall.

Therefore, retinal arteriolar and venular changes in diameter in response to flickering light are calculated automatically by the DVA software (after averaging the two obtained examinations for each eye) and reported as percentage (%) in comparison with baseline diameter. During DVA testing, healthy eyes are characterized by a characteristic response curve to flicker light: primary vasodilation and secondary vasoconstriction. The main outcome measures of this dynamic analysis were: (i) arterial dilation; (ii) arterial constriction; (iii) reaction amplitude (the difference between arterial dilation and constriction); and (iv) venous dilation (Fig. 1).

In addition, the DVA software provided other secondary outcomes, which are dependent on the baseline assessment of the retinal vessels (static analysis). These parameters are calculated by measuring the average diameter of all those arterioles and venules within an annulus around the optic disc. The obtained parameters are, as follows: (i) central retinal artery equivalent (CRAE); (ii) central retinal vein equivalent (CRVE); and (iii) artery-vein ratio (AVR – obtained by dividing CRVE by CRAE).

### Optical Coherence Tomography Angiography

All enrolled subjects underwent two OCTA successive scans using OCTA software (Cirrus 5000 with Angioplex™; Carl Zeiss Meditech) and covering 3 × 3-mm and 6 × 6-mm area centered on the fovea, respectively.

The main OCTA outcome measures were: (i) superficial vascular plexus (SVP) perfusion density; (ii) deep vascular plexus (DVP) perfusion density; (iii) choriocapillaris (CC) perfusion density; and (iv) choroidal perfusion density.

An experienced grader (RS), masked for subject information, checked the quality of all images and only eyes with OCTA images of sufficient quality were included in the image processing. For each eye, we exported the SVP, DVP, CC and choroid *en face* OCTA scans, which were automatically segmented by the Angioplex™ software, as previously described^26,27^. The three *en face* images were then imported into image analysis ImageJ software version 1.50 (National Institutes of Health, Bethesda, MD; available at http://rsb.info.nih.gov/ij/index.html)^28^.

To calculate the perfusion density, a previously described thresholding algorithm was applied to the *en face* images to create binary images^29,30^. In brief, the images were first converted from RGB to 8-bit. Then, the images were binarized using the “mean” thresholding method. The perfusion density was thus calculated as a proportion of the number of pixels over the threshold divided by the total number of pixels in the analyzed area. Of note, in the SVP and DVP measurements, the region corresponding to the foveal avascular zone (FAZ) was masked.

### Optical Coherence Tomography

The SPECTRALIS HRA + OCT device (Heidelberg Engineering, Heidelberg, Germany) was used for the spectral domain OCT examination. The protocol of acquisition consisted in 19 B-scan sections centered on the fovea. This acquisition allowed the software to automatically segment the retinal layers and produce a thickness map for each of them^31^.

The algorithm identified and calculated the difference between the inner limiting membrane (ILM) and the retinal pigment epithelium (RPE), and yielded the average macular thickness. Moreover, the algorithm provided the average thickness of the following slabs: (i) retinal nerve fiber layer (RNFL); (ii) ganglion cell layer (GCL); (iii) inner plexiform layer (IPL); and (iv) ganglion cell complex (GCC - resulted as GCL + IPL). The software provided the average sectorial thicknesses (superior, inferior, nasal, temporal) in two different rings centered on the fovea (dimensions: inner and outer diameters of 3 and 6 mm, respectively). In addition, the central thickness is referred to the average thickness in the 1-mm diameter central circle.

### Statistical analysis

Statistical calculations were performed using Statistical Package for Social Sciences (version 20.0, SPSS Inc., Chicago, IL, USA).

To detect departures from normal distribution, a Shapiro-Wilk’s test was performed for all variables. Means and standard deviation (SD) were computed for all quantitative variables. Continuous variables were compared by conducting a Student T-test for independent variables or a one-way analysis of variance (ANOVA) with Bonferroni post-hoc test. Statistical significance of the differences between groups for qualitative variables was assessed using Fisher’s exact test. Pearson’s chi-squared correlation was performed to evaluate the linear correlation between the CSF level of Aβ, tau and phosphorylated-tau proteins and functional/anatomic metrics. The latter test was performed by considering together AD and MCI subjects. A p value of 0.05 was considered for statistical significance.

## Results

### Characteristics of subjects included in the analysis

Twelve subjects with AD (8 females), 12 subjects with MCI (7 females), and 32 gender- and age-matched controls (15 females) were included in the analysis (ANOVA for difference in gender: p = 0.469). One eye for each subject was randomly selected and included in the analysis.

Mean age was 72.9 ± 7.2 years [range 58–81 years] in the AD group, 76.3 ± 6.9 years [range 59–87 years] in the MCI group, and 71.6 ± 5.9 years [range 45–84 years] in the control group (p = 0.104). The BCVA was 0.2 ± 0.2 LogMAR (Snellen VA of 20/32) in the AD group, 0.1 ± 0.1 LogMAR (Snellen VA of 20/25) in the MCI group, and 0.1 ± 0.1 LogMAR (Snellen VA of 20/25) in the healthy eyes (p = 0.090).

Neurological characteristics of AD and MCI subjects are described in Table 1.

**Table 1:** Neurological parameters in affected subjects.

	MCI	AD
MMSE, mean ± SD*	24.9/30 ± 2.7/30	20.7/30 ± 3.6/30
Corrected MMSE, mean ± SD*	23.7/30 ± 2.4/30	19.5/30 ± 3.6/30
CSF - Amyloid β (ng/L), mean ± SD	514.55 ± 182.75	486.90 ± 100.91
Negative (%)	50.0	66.7
Positive (%)	50.0	33.3
CSF - Tau protein (ng/L), mean ± SD	515.27 ± 364.14	527.30 ± 298.35
Negative (%)	75.0	44.4
Positive (%)	25.0	55.6
CSF - Phosphorylated tau (ng/L), mean ± SD^†^	81.91 ± 41.90	83.90 ± 49.91
Negative (%)	75.0	22.2
Positive (%)	25.0	77.8
Arterial hypertension		
Absent (%)	54.5	36.4
Under treatment (%)	36.4	63.6
Bad controlled (%)	9.1	0
Cerebrovascular disease		
Negative (%)	36.4	54.5
Positive (%)	63.6	45.5

*T-student test p value is < 0.05; ^†^Fisher’s exact test p value is <0.05.

MCI: mild cognitive impairment; AD: Alzheimer’s disease; MMSE: mini-mental state examination; SD: standard deviation; CSF: cerebrospinal fluid; ^18^FDG PET: ^18^fluorodeoxyglucose positron emission tomography.

### Functional metrics – Dynamic Vessel Analyzer

**Figure 2:**
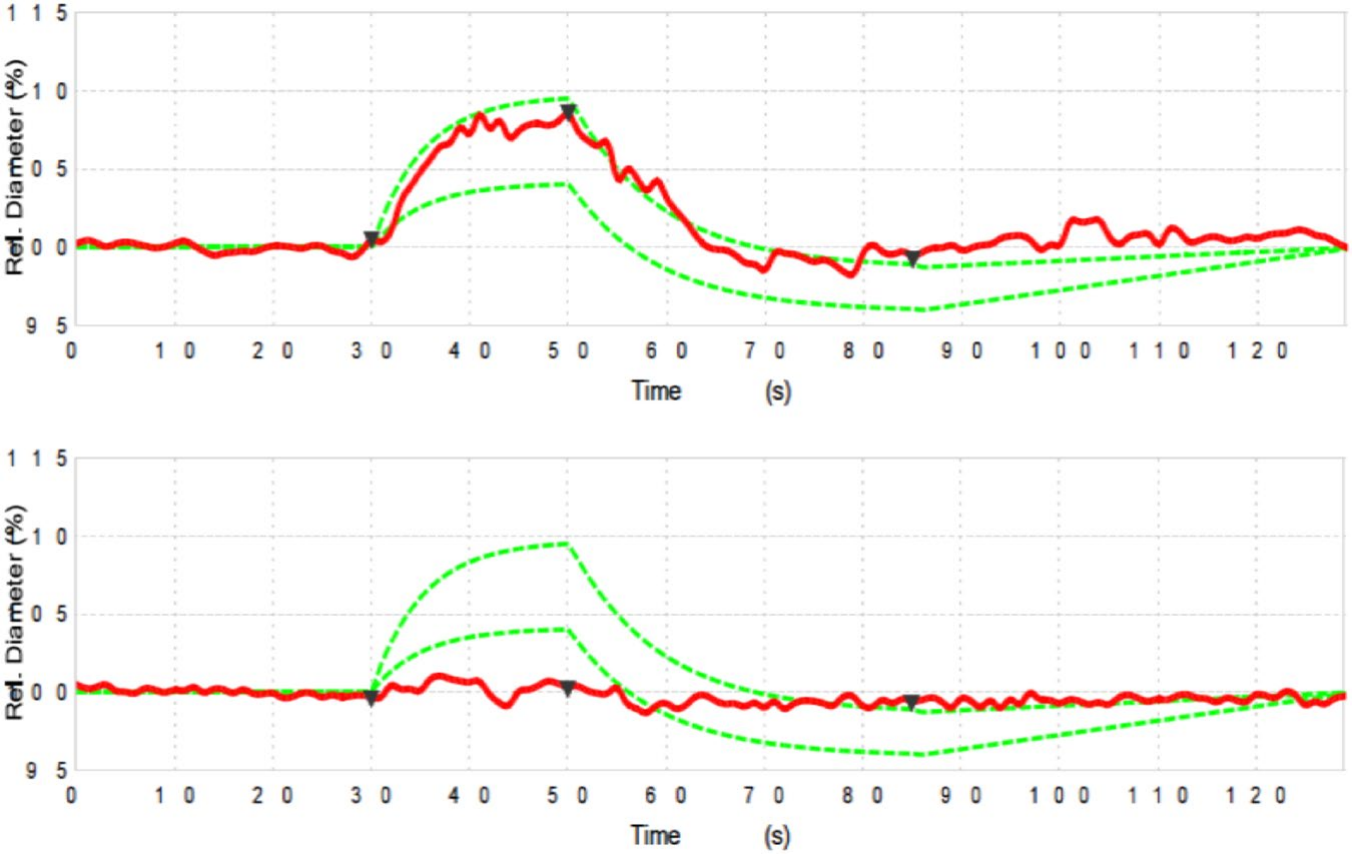
Representation of Alzheimer’s and healthy cases. Example of dynamic vessel analyzer curve in a healthy control (top image) and in an Alzheimer’s subject (bottom image). In the affected subject, a loss of dilation and constriction, as well as an overall decrease in amplitude, indicate significant dysfunction of the response to flicker stimulus.

Using DVA, we investigated both vascular static and dynamic changes in AD and MCI subjects. In the static analysis, none of the analyzed parameters (AVR, CRAE, and CRVE) differed among groups (Table 2). In the dynamic analysis, the arterial dilation was decreased in the AD group (0.77 ± 2.06%), in the comparison with both the MCI (2.84 ± 2.18%, p = 0.045) and control (3.53 ± 1.25%, p = 0.002) groups. Neither arterial constriction nor venous dilation differed among groups. The reaction amplitude was decreased both in AD (0.21 ± 1.80%, <0.0001) and MCI (2.29 ± 1.81%, p = 0.048) subjects, compared to controls (3.86 ± 1.94%) (Table 2, Fig. 2).

**Table 2:** Dynamic vessel analyzer parameters in affected subjects and controls.

	AD	MCI	Controls
**Dynamic Analysis**			
*Arterial dilation (%)*	0.77 ± 2.06	2.84 ± 2.18	3.53 ± 1.25
		0.045^*^	0.002^*^
	—		1.0^†^
*Arterial constriction (%)*	0.59 ± 0.88	0.55 ± 1.28	−0.33 ± 1.17
		1.0^*^	0.163^*^
			0.199^†^
*Reaction amplitude (%)*	0.21 ± 1.80	2.29 ± 1.81	3.86 ± 1.94
		0.038^*^	<0.0001^*^
			0.048^†^
*Venous dilation (%)*	2.84 ± 1.56	3.45 ± 1.96	3.37 ± 1.81
		1.0^*^	0.833^*^
			1.0^†^
**Static Analysis**			
*AVR*	0.92 ± 0.10	0.83 ± 0.10	0.89 ± 0.06
		0.070^*^	1.0^*^
	—		0.236^†^
*CRAE*	189.81 ± 25.18	177.94 ± 26.92	186.77 ± 23.63
		0.924^*^	1.0^*^
			1.0^†^
*CRVE*	208.04 ± 28.31	213.78 ± 16.60	207.31 ± 16.53
		1.0^*^	1.0^*^
			1.0^†^

^*,†^ANOVA test with Bonferroni post-hoc *Comparison versus AD; ^†^Comparison versus MCI. Data are presented as mean ± SD (standard deviation).

AD: Alzheimer’s disease; MCI: mild cognitive impairment; AVR: artery-vein ratio; CRAE: central retinal artery equivalent; CRVE: central retinal vein equivalent.

### Anatomic metrics

**Figure 3:**
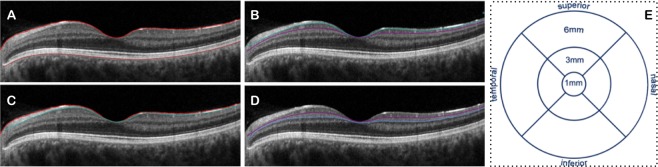
OCT scan from a healthy subject. The average macular thickness was measured as the difference between the inner limiting membrane (ILM) and the retinal pigment epithelium (RPE) (image **A**). Moreover, the algorithm provided the average thickness of the ganglion cell layer (GCL – image **B**), retinal nerve fiber layer (RNFL – image **C**), and inner plexiform layer (IPL – image **D**). (**E**) The build-in software provided the sectorial thicknesses (superior, inferior, nasal, temporal) in two different rings centered on the fovea (dimensions: inner and outer diameters of 3 and 6 mm, respectively). In addition, the central thickness was referred to the average thickness in the 1-mm diameter central circle.

Optical coherence tomography was carried out to assess GCL thickness in enrolled subjects, since neuroretinal changes have been demonstrated to occur in AD and MCI subjects^32–35^. Notably, the average GCL thickness was reduced in AD subjects compared to controls, but only in the central (12.59 ± 2.60 µm versus 15.02 ± 2.74 µm, p = 0.025) and temporal (42.05 ± 4.95 µm versus 47.02 ± 5.27 µm, p = 0.040) sectors (Table 3, Fig. 3).

**Table 3:** Optical coherence tomography parameters in affected subjects and controls.

	Sector	AD	MCI	Controls
Average RNFL thickness (µm)	Central 1 mm*	12.18 ± 1.66	11.80 ± 3.16^†^	13.50 ± 1.36
	Superior 3 mm	23.27 ± 3.55	23.40 ± 1.97	23.89 ± 3.08
	Temporal 3 mm	17.82 ± 3.08	16.95 ± 0.76	17.35 ± 1.58
	Inferior 3 mm	24.05 ± 2.53	23.10 ± 2.25	24.52 ± 3.31
	Nasal 3 mm	19.41 ± 2.71	20.00 ± 2.30	20.74 ± 1.84
	Total 3 mm	21.18 ± 2.21	20.84 ± 1.19	21.61 ± 1.88
	Superior 6 mm	36.55 ± 8.35	35.70 ± 3.46	35.18 ± 4.50
	Temporal 6 mm	20.05 ± 1.85	19.20 ± 0.75	19.52 ± 1.62
	Inferior 6 mm	36.30 ± 8.17	36.25 ± 5.53	36.70 ± 7.33
	Nasal 6 mm	48.27 ± 8.89	47.15 ± 5.08	48.87 ± 8.06
	Total 6 mm	35.15 ± 6.36	34.44 ± 3.24	34.88 ± 5.01
Average GCL thickness (µm)	Central 1 mm*	12.59 ± 2.60^†^	12.95 ± 2.70	15.02 ± 2.74
	Superior 3 mm	47.59 ± 5.27	51.64 ± 6.33	51.93 ± 4.21
	Temporal 3 mm*	42.05 ± 4.95^†^	46.32 ± 7.31	47.02 ± 5.27
	Inferior 3 mm	47.82 ± 3.82	50.73 ± 5.90	52.41 ± 4.61
	Nasal 3 mm	47.32 ± 4.47	48.05 ± 7.47	50.50 ± 4.98
	Total 3 mm	46.24 ± 4.45	49.11 ± 6.38	50.47 ± 4.45
	Superior 6 mm	34.15 ± 4.35	36.36 ± 4.48	35.95 ± 3.64
	Temporal 6 mm	32.82 ± 4.85	34.82 ± 5.43	33.59 ± 4.84
	Inferior 6 mm	34.20 ± 4.52	36.23 ± 4.61	35.30 ± 4.21
	Nasal 6 mm	35.09 ± 3.65	36.73 ± 4.59	37.15 ± 3.70
	Total 6 mm	34.47 ± 4.16	36.04 ± 4.27	35.32 ± 3.59
Average IPL thickness (µm)	Central 1 mm	19.50 ± 2.52	19.55 ± 3.11	20.54 ± 2.62
	Superior 3 mm	39.27 ± 3.51	41.36 ± 3.22	41.30 ± 3.22
	Temporal 3 mm*	38.68 ± 3.17^†^	40.55 ± 3.78	41.93 ± 3.17
	Inferior 3 mm	39.09 ± 2.82	40.59 ± 3.65	40.89 ± 3.21
	Nasal 3 mm	40.09 ± 2.98	41.05 ± 3.88	42.91 ± 3.48
	Total 3 mm	39.36 ± 2.80	40.89 ± 3.49	41.76 ± 3.00
	Superior 6 mm	28.20 ± 3.08	29.77 ± 3.66	29.86 ± 2.77
	Temporal 6 mm	30.86 ± 2.84	31.23 ± 4.09	31.13 ± 3.15
	Inferior 6 mm	28.30 ± 3.17	29.91 ± 3.82	29.00 ± 3.36
	Nasal 6 mm	27.50 ± 3.08	29.14 ± 3.43	29.00 ± 2.99
	Total 6 mm	29.15 ± 2.35	29.96 ± 3.56	29.66 ± 2.74
Average GCC thickness (µm)	Central 1 mm	32.09 ± 4.95	32.50 ± 5.55	35.57 ± 5.13
	Superior 3 mm	86.86 ± 8.22	93.00 ± 9.36	93.24 ± 7.23
	Temporal 3 mm*	80.73 ± 7.93^†^	86.86 ± 10.84	88.96 ± 7.99
	Inferior 3 mm	86.91 ± 6.36	91.32 ± 9.46	93.30 ± 7.59
	Nasal 3 mm	87.41 ± 7.10	89.09 ± 11.10	93.41 ± 8.11
	Total 3 mm	85.61 ± 6.97	90.00 ± 9.74	92.23 ± 7.27
	Superior 6 mm	62.35 ± 7.26	66.14 ± 8.10	65.82 ± 6.29
	Temporal 6 mm	63.68 ± 7.45	66.05 ± 9.32	64.72 ± 7.77
	Inferior 6 mm	62.50 ± 7.63	66.14 ± 8.28	64.30 ± 7.44
	Nasal 6 mm	62.59 ± 6.66	65.86 ± 7.93	66.15 ± 6.44
	Total 6 mm	63.62 ± 6.41	66.00 ± 7.78	64.98 ± 6.20
Average macular thickness (µm)	Central 1 mm	268.41 ± 17.98	261.30 ± 26.36	278.17 ± 15.78
	Superior 3 mm*	328.95 ± 11.69^†^	338.15 ± 17.18	342.89 ± 16.70
	Temporal 3 mm	319.86 ± 9.76	324.45 ± 21.11	331.43 ± 16.19
	Inferior 3 mm	330.09 ± 11.56	334.45 ± 20.52	341.52 ± 15.54
	Nasal 3 mm*	333.32 ± 12.48^†^	334.20 ± 20.11	347.22 ± 15.41
	Total 3 mm	328.04 ± 10.80	332.69 ± 19.18	340.81 ± 15.49
	Superior 6 mm	292.85 ± 8.86	299.60 ± 17.13	300.68 ± 16.09
	Temporal 6 mm	275.05 ± 9.36	277.80 ± 16.46	278.46 ± 15.52
	Inferior 6 mm	286.50 ± 13.01	293.10 ± 18.50	292.38 ± 16.67
	Nasal 6 mm	304.77 ± 14.58	306.60 ± 17.00	312.35 ± 17.17
	Total 6 mm	290.04 ± 11.08	294.14 ± 16.77	295.85 ± 15.37

*ANOVA p value is < 0.05; ^†^Statistically significant different compared to control. Data are presented as mean ± SD (standard deviation).

AD: Alzheimer’s disease; MCI: mild cognitive impairment; RNFL: retinal nerve fiber layer; GCL: ganglion cell layer; IPL: inner plexiform layer; GCC: ganglion cell complex (GCC is equivalent to GCL + IPL).

**Figure 4:**
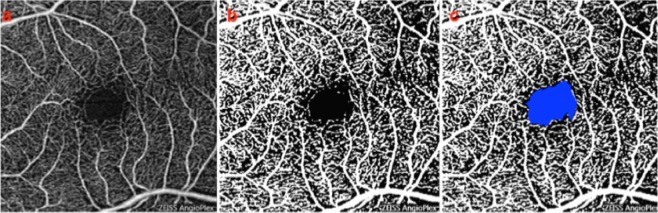
OCTA images from a healthy subject. (**a**) The image of the superficial vascular plexus (SVP) was first imported in image analysis ImageJ software (National Institutes of Health; http://imagej.nih.gov/ij/)^28^. This image was then binarized with a “mean” method (**b**). The perfusion density was thus calculated as a unitless proportion of the number of pixels over the threshold (white area in the **b** and **c** images)) divided by the total number of pixels in the analyzed scan area. Of note, the region of the foveal avascular zone (blue in image **c**) was masked before measuring the perfusion density.

No differences in OCTA parameters were found among groups in either 3 × 3 and 6 × 6 scan (Table 4, Fig. 4). This was in disagreement with the Bulut and colleagues^36^ paper (see Discussion section).

**Table 4:** Optical coherence tomography angiography parameters in affected subjects and controls.

Scan	Vascular layer	AD	MCI	Controls
3 × 3-mm	SVP (%)	40.66 ± 2.36	42.13 ± 1.26	40.92 ± 1.96
	DVP (%)	43.78 ± 1.00	45.05 ± 1.26	45.77 ± 1.77
	Choriocapillaris (%)	46.21 ± 0.80	45.94 ± 1.55	46.16 ± 1.12
	Choroid (%)	45.94 ± 1.74	46.21 ± 1.12	46.10 ± 1.07
6 × 6-mm	SVP (%)	40.43 ± 1.32	40.70 ± 2.76	40.31 ± 1.93
	DVP (%)	44.36 ± 1.63	44.49 ± 0.76	44.82 ± 1.41
	Choriocapillaris (%)	46.36 ± 0.34	45.87 ± 1.15	46.65 ± 1.11
	Choroid (%)	46.96 ± 1.97	45.77 ± 1.63	46.22 ± 0.96

Data are presented as mean ± SD (standard deviation).

AD: Alzheimer’s disease; MCI: mild cognitive impairment; SVP: superficial vascular plexus; DVP: deep vascular plexus; SD, standard deviation; *ANOVA p value < 0.05; ^#^Statistically significant different if compared to control.

### Correlation analysis

In the Pearson correlation analysis, the CSF amyloid β level was directly correlated with the arterial dilation (R = 0.441, p = 0.040) and reaction amplitude (R = 0.580, p = 0.005). Furthermore, the CSF amyloid β amount was directly correlated with the central GCL average thickness (R = 0.530, p = 0.011) and the central IPL average thickness (R = 0.497, p = 0.019). The central IPL average thickness was inversely correlated with the CSF phosphorylated-tau quantity. Other significant correlations are reported in Table 5.

**Table 5:** Pearson correlations reaching statistical significance.

	Aβ		tau		phosphorylated tau	
	r	p-value	r	p-value	r	p-value
Arterial dilation	0.441	0.040				
Reaction amplitude	0.580	0.005				
CRAE					0.424	0.049
CRVE	−0.429	0.046	0.470	0.027	0.519	0.013
Central GCL average thickness	0.530	0.011				
Central IPL average thickness	0.497	0.019			−0.437	0.042
Central GCC average thickness	0.527	0.012			−0.440	0.041
Central Macular thickness	0.453	0.039				

Aβ: amyloid β; CRAE: central retinal artery equivalent; CRVE: central retinal vein equivalent; GCL: ganglion cell layer; IPL: inner plexiform layer; GCC, ganglion cell complex (GCL + IPL).

## Discussion

In this cross-sectional study we investigated quantitative data of retinal vessels in eyes of subjects with Alzheimer’s disease or mild cognitive impairment. Overall we found that subjects with AD are characterized by a significant impairment of the retinal neurovascular coupling. Noteworthy, results of the present study demonstrated that similar alterations feature subjects with MCI.

Although AD is thought to be a disorder which mainly affects the brain, several evidences suggest that the retina may be affected^35,37–42^. Several approaches have been used to demonstrate that the degeneration of the retinal ganglion cells is associated with AD, perhaps offering an effective measure of neurodegenerative progression^37–39^. Histopathological studies analyzed the postmortem retinas from subjects with AD and demonstrated a reduction in the number of the retinal ganglion cells and their axons in the nerve fiber layer^37–39^. This cell loss has been speculated to be secondary to the presence of extracellular plaques and intracellular Aβ deposits in the retina of subjects with Alzheimer’s disease^40^. Different studies have used OCT to demonstrate that the GCL thickness is reduced in AD populations^32–35,41,42^.

Our results similarly highlighted that eyes of subjects with AD, but not with MCI, are characterized by a thinning of the GCL. Importantly, we found that this thinning is mainly confined to the central and temporal regions. These results are consistent with previous histopathological studies, since in the central 0–2 mm region of the retina 97% of neurons in the GCL are ganglion cells (while the remaining 3% consist of displaced amacrine cells)^43^. Furthermore, AD is known to mainly affect the largest class of retinal ganglion cells (M-cells)^38^, whose amount is greater in the temporal macula.

Even though neuronal alterations are known to be predominant in both AD and MCI, evidences from neuropathology and neuroimaging studies demonstrated that cerebral microvascular changes are associated with these disorders^8,44–47^. Although the mechanisms driving this association remain elusive, one proposed process regards amyloid β, which was demonstrated to be deposited in and around cerebral blood vessels^48^. While there is evidence that cerebral vessels may be affected in subjects with these neurodegenerative disorders, these vessels remain difficult to assess *in vivo* and their use as indicator of diagnosis and disease progression is therefore limited. For this reason, an increased interest in the retinal vessels, which may be considered a surrogate for cerebral microcirculation, has led to several histopathological and imaging studies showing that retinal vessels may be affected in AD and MCI^11,12,49^.

We add to the literature by reporting the functional response of retinal vessels in a population of subjects affected by these two forms (or consecutive phases) of cognitive impairment. During DVA testing, two components of a characteristic response curve to flicker light impulse may be identified: primary vasodilation and secondary vasoconstriction. The vasodilatation is thought to follow the stimulation of the photoreceptors, which is followed by an increased retinal blood flow. The process mediating this response is known as neurovascular coupling and presence and amplitude of a typical, biphasic response are established indicators for autoregulatory quality^50^. In our AD cohort, we observed an overall reduction of vessel response, which mostly affected the vasodilatation of the arteries. Since the cerebral and retinal blood flow was demonstrated to be decreased in AD^51^, we suppose that this chronic hypoperfusion could impair the endothelial function and the production of nitric oxide (NO), which is vital for the vasodilatation process. Alternatively, the reduced response may be due to an augmented rigidity of the retinal arteries, in view of the fact that the brain vessels were demonstrated to be characterized by a thickened basal membrane in AD subjects^52^. Finally, the decreased vascular response might be secondary to neuronal degeneration, which may cause an overall diminished metabolic demand.

In our study, DVA analysis showed that even MCI subjects are characterized by an alteration of the vascular response to flicker stimulus. These results may suggest that these functional alterations are early in the pathogenesis and that precede the retinal neuronal loss. Since MCI have an increased risk of developing AD, there is significant interest in the clinical characterization of these subjects and in finding new clinical parameters which may help in the diagnosis and eventually predict the transition from MCI to AD. If replicated in future studies, DVA analysis may prove to be a useful parameter for characterizing subjects with cognitive impairment. Furthermore, a prospective longitudinal evaluation of retinal vessels in MCI subjects might clarify if retinal vascular assessment may be used to predict clinical progression.

Our results are in disagreement with those shown by Kotliar and colleagues^53^, who demonstrated an increase arterial vasodilation in both AD and MCI groups. However, in the Kotliar *et al*.’s study the enrolled subjects did not undergo an ophthalmological evaluation before enrollment and this might have affected their results, since several ocular diseases are known to affect the DVA results^14,24,25^.

Notably, considering both AD and MCI subjects, we found that cerebrospinal level of amyloid β directly correlated with the amount of arterial dilation after flicker (increased CSF Aβ amount is thus correlated with a better neurovascular coupling). The amyloid load and the presence of neurofibrillary pathologic abnormalities in the brain are known to be inversely correlated with Aβ CSF quantity, and directly correlated with CSF phosphorylated-tau level^19^. Therefore, taken these data together, we may speculate that an increased pathological accumulation of proteins in the brain and in the retina may directly affect the retinal neurovascular coupling, with one of the mechanisms discussed above.

In addition to the functional assessment of the retinal vessels, our study cohort was investigated with OCTA, which provides a morphological characterization of the retinal and anterior choroidal vessels without the need for dye injection. Moreover, OCTA has the additional advantage of depth-resolution with better visualization of the deeper vascular layers, with consequent improved vascular characterization in several ocular and systemic disorders, including other neurodegenerative diseases^54^. Bulut *et al*.^36^ first investigated AD subjects using OCTA. In the latter study, the authors enrolled 26 AD subjects and 26 age-matched healthy controls and demonstrated that AD eyes are characterized by a reduced retinal vascular density. Of note, we demonstrated that AD subjects are characterized by a slightly reduction in flow in the deep vascular plexus, which however did not reach statistical significance. These results seem to suggest that functional alterations of the retinal vessels precede their morphological changes. Further studies with larger sample size are required to address this important point.

The main limitation of our study is the employment of a single time point for each patient. A prospective longitudinal evaluation of retinal vessels in AD and MCI subjects will shed further light on the role of the vascular damage on the structural alterations of the retina. Another limitation is the small sample size of affected subjects, which makes it difficult to draw conclusions to the overall AD or MCI population.

In conclusion, this is the first fully integrated report of the retinal vessels in subjects with either AD or MCI. We demonstrated that vascular changes characterize these disorders and that such alterations are mainly limited to the arterial function, rather than a vascular loss. Future studies with extended longitudinal follow up of the studied cohort may provide additional substantive information and retinal vascular parameters may prove to be a useful biomarker for monitoring the efficacy of AD and MCI, or to predict the disease progression.
